# Late Nutrition and Negative Outcomes in a Brazilian Paediatric Intensive Care Unit: A Retrospective Cohort

**DOI:** 10.1111/jpc.70049

**Published:** 2025-04-05

**Authors:** Arnildo Linck Júnior, Flávia Lopes Gabani, Edmarlon Girotto, Ana Maria Rigo Silva, Selma Maffei de Andrade

**Affiliations:** ^1^ Department of Pediatrics and Pediatric Surgery State University of Londrina (UEL) Paraná Brazil; ^2^ Department of Nursing State University of Londrina (UEL) Paraná Brazil; ^3^ Pharmaceutical Sciences Department State University of Londrina (UEL) Paraná Brazil; ^4^ Public Health Department State University of Londrina (UEL) Paraná Brazil

**Keywords:** enteral nutrition, healthcare‐related infections, hospital mortality, length of stay, paediatric intensive care units

## Abstract

**Aim:**

To analyse the association between late initiation of enteral nutrition and negative outcomes in the Paediatric Intensive Care Unit (PICU) in Brazil.

**Method:**

This was a retrospective cohort study with data from the medical records of children hospitalised between 2012 and 2017. The independent variable was late initiation of enteral nutrition (> 24 h after admission). The outcomes analysed included longer length of stay in the PICU and hospital, incidence of healthcare‐related infections (HAIs), and death. Poisson regression models with robust variance were adjusted for potentially confounding variables by presenting relative risks (RR) and 95% confidence intervals (95% CI).

**Results:**

We analysed 840 hospitalizations: 311 (37.0%) with delayed initiation of nutrition, 252 (30.0%) with a diagnosis of HAIs, and 93 deaths (11.1%). After all adjustments, late initiation of enteral nutrition was associated with longer lengths of stay in the PICU (RR: 1.41; 95% CI: 1.01–1.30), hospital stay (RR: 1.22; 95% CI: 1.06–1.41), and higher HAI incidence (RR: 1.40; 95% CI: 1.14–1.73). The association with mortality was no longer significant after adjusting for the admission severity indicators.

**Conclusion:**

The results indicate that late initiation of nutrition can lead to longer PICU and hospital stays and a higher incidence of HAIs.

**Summary**

- What is already known on this topic
  ○ The critically ill child is exposed to situations of metabolic stress, with compromised nutritional status.
  ○ It is believed that the longer the fasting time, the worse the patient's clinical outcomes, especially the functional impairment of the intestine and exposure of the body to nosocomial infections.
  ○ The early introduction of enteral nutrition aims to reduce the morbidity and mortality of children admitted to the Paediatric Intensive Care Unit.
- What this study adds
  ○ The present study found a correlation between fasting for more than 24 h and longer stays in the Paediatric Intensive Care Unit and hospital.
  ○ An association between received delayed nutrition and higher incidence of infections related to health care was also observed.
  ○ There was no significant difference in death incidence between those who received delayed nutrition and those who did not in the adjusted analysis.

## 1 Introduction

Severe diseases in the Paediatric Intensive Care Unit (PICU) are associated with inflammatory and endocrine changes that promote tissue catabolism and are characterised by high energy consumption, particularly in infants [1]. These diseases, which are associated with late initiation of nutritional support, may result in reduced muscle mass, weight loss and malnutrition [2, 3].

A delay in starting nutritional support may also increase intestinal permeability, favouring bacterial translocation and development of new infections [4]. These events result in prolonged antimicrobial use and hospitalisation, increasing morbidity and mortality in debilitated patients [5, 6].

The early introduction of enteral nutrition aims to reduce the morbidity and mortality of children admitted to the PICU [7]. It is defined as the initiation of feeding in the first 24 h by some authors [8], and 48 or 72 h by others [9]. This approach ensures the necessity of a protein energy substrate to prevent or mitigate damage caused by exacerbated catabolism [10]. Thus, the earlier the initiation of nutrition, the faster the nutritional goals of critically ill patients are achieved [7].

Most studies on negative outcomes related to late initiation of nutrition in the PICU are concentrated in developed countries. They involve paediatric hospitalisations in specific situations such as victims of traumatic brain injury (TBI), post‐surgery patients, or patients with metabolic disorders [4]. Thus, this study aimed to analyse the association between late initiation of enteral nutrition and negative outcomes in children with a mixed profile of causes of hospitalisation in a PICU in Brazil.

## 2 Materials and Methods

### 2.1 Study Design and Location

This was a retrospective cohort study with data from hospitalisations of children admitted to the PICU of a highly complex teaching hospital in southern Brazil from January 1, 2012, to December 31, 2017. The PICU admits approximately 200 children each year in the age range of zero to 17 years, with a mixed profile of diseases and postoperative cases of paediatric surgeries.

### 2.2 Research Instrument

A standardised form was used for data collection, with fields filled in regarding sociodemographic characteristics, personal and family background, and admission and hospitalisation conditions, including information on ventilatory support instituted for the patients, need for vasoactive drugs and death.

### 2.3 Data Collection

Data were collected from medical records, and the forms were completed by two professors from the departments of paediatrics and paediatric surgery and nursing, and by undergraduate and graduate students. The students underwent training, which consisted of a presentation of the data collection instrument and guidance on how to search for information in the medical records. After students completed the forms, they were reviewed by their professors. The collection was extended to December 31, 2019, providing each hospitalisation with 24 months of follow‐up to observe the outcomes analysed. After collection and verification, the data were entered into a database created using the public domain programme Epi Info version 3.5.4.

### 2.4 Study Variables

We excluded hospitalisations that lasted less than 24 h, those whose outcome was death before starting enteral nutrition, and those who remained hospitalised for more than 24 months (the maximum follow‐up time). The main independent variable was the introduction of enteral nutrition, categorised as early nutrition (starting ≤ 24 h) or late nutrition (> 24 h). The outcomes analysed were the length of PICU stay, length of hospital stay, diagnosis of healthcare‐associated infections (HAIs) and death. The length of stay in the PICU was calculated as the difference between the discharge and admission dates in days and then categorised by the median (≤ 5 days; > 5 days). The same procedure was performed for the length of hospital stay, calculated first in days, and then categorised by median (≤ 13 days; > 13 days). For the diagnosis of HAIs, only infections recognised 48 h after admission were considered, with the information provided in medical records or through the results of tracheal secretion cultures, when the isolated etiological agent was compatible with the clinically suspected infection, considering the colony size greater than 100 000/mL.

Other variables investigated as potential confounders for adjustment of the analyses were age (< 28 days, 28 days to < 1 year, 1 year to < 4 years, 4 years to < 7 years, 7 years or older), sex (male or female), the child's city of origin (hospital host municipality; other municipalities), diagnosis of chronic disease and malnutrition at admission, need for ventilatory assistance or a vasoactive drug in the first 24 h after admission, and probability of death in the first 24 h of hospitalisation. Chronic disease diagnosis was categorised as “yes” when there was persistence of the same signs or symptoms specific to one or more organs or systems for 30 days or more before admission. Nutritional status was classified using the *z*‐weight‐for‐age score, and children with scores lower than –2 were considered malnourished. The need for ventilatory assistance was defined when the child remained connected to the mechanical respirator, either invasively (orotracheal intubation or tracheostomy) or non‐invasively through nasal or facial interfaces, in modalities described as Continuous Positive Airway Pressure (CPAP) or Bi‐level Positive Airway Pressure (BiPAP). The need for vasoactive drugs was defined as the use of drugs to increase blood pressure and improve cardiac output. To assess the probability of death in the first 24 h of hospitalisation, the Clinical Risk Index for Babies (CRIB) was used for children up to 27 days of life and the Paediatric Risk of Mortality, Version II (PRISM‐II) for those older than 27 days, in which the respective scores obtained by these scores were reclassified into percentages corresponding to the risk of death, with a theoretical possibility of variation from 0% to 100% [11].

Sex and age were used as variables because physiology can have an impact on the evolution of certain diseases, particularly when children under 1 year of age are analysed. In the case of the municipality of origin, it is believed that the availability of resources, as well as the type and conditions of transportation, may interfere with the clinical conditions of admission. Complex chronic conditions, such as malnutrition, directly compromise the child's clinical condition, regardless of the diagnosis or severity of the current illness. Regarding the need for mechanical ventilation, it was considered that the need for ventilatory assistance may independently indicate the severity of the admission diagnosis. The analysis of children who required ventilatory assistance, whether invasive or non‐invasive, was carried out dichotomously, without distinguishing the method used for artificial ventilation. To calculate the PRISM II score, clinical variables were used, such as blood pressure, heart and respiratory rate, pupillary reactions, and the Glasgow Coma Scale, and laboratory variables, such as partial pressure of carbon dioxide, relationship between partial pressure of oxygen and inspired fraction offered, coagulogram and serum levels of bilirubin, calcium, glucose, bicarbonate and potassium. To measure the CRIB score, birth weight, gestational age, congenital malformations, base excess, and fraction of inspired oxygen were scored.

### 2.5 Data Analysis

The analyses were performed using Poisson multiple regression with robust variance, crude and adjusted relative risks (RR), and respective 95% confidence intervals (95% CI). Adjustments were made for each outcome using two progressive models that initially included demographic variables and diagnoses of chronic disease and malnutrition (Model 1). Subsequently, adjustments were made to indicate the severity of the child's condition during hospitalisation: the need for ventilatory assistance or vasoactive drugs and the probability of death based on CRIB or PRISM‐II scores (Model 2). The variables that presented *p*‐values < 0.20 in the crude analysis were included in the adjusted models, except for the child's age, which was included in all models regardless of the p‐value. The Kaplan–Meier curve was used to analyse each outcome over time (hospitalisation hours in the PICU), considering late initiation of enteral nutrition as a factor, with comparisons between groups performed using the Log‐Rank (Mantel–Cox) test. The data were analysed using the Statistical Package for Social Sciences (SPSS) version 19.0.

## 3 Results

### 3.1 Characterisation of the Study Population

During the study period, 1223 hospitalizations were identified, 41 of which were not located and 71 did not contain information on enteral nutrition initiation. Thus, data from 1111 admissions (90.8%) were initially analysed. Seventy‐two patients were excluded because death occurred before starting enteral nutrition, and 197 because the length of stay was less than 24 h. Two hospitalisations were excluded because they lasted for longer than 24 months. Therefore, 840 hospitalisations were included in the analysis. Of these, 311 (37.0%) children received enteral nutrition after 24 h of hospitalisation. Children who died before the start of feeding usually had digestive malformations, such as oesophageal atresia, intestinal atresia and gastroschisis, and who suffered injuries resulting from infections or the association with other malformations, such as congenital heart disease. Most of these children died within the first seven days of hospitalisation.

The age of the study population ranged from zero to 178 months, with a median of 11 months (interquartile range:1–57 months). Approximately one‐fifth (20.7%) were newborns, 30.2% were infants, and 71.9% were under four years of age (Table 1). More than half (53.2%) were male, 62.6% were from other municipalities, 46.1% had a diagnosis of chronic illness, and 28.1% were considered malnourished. Most patients required ventilatory assistance in the first 24 h of hospitalisation (70.1%), and almost a quarter (23.8%) required drugs with vasoactive action during the same period.

**TABLE 1:** Demographic and clinical characteristics of Paediatric Intensive Care Unit (PICU) admissions, 2012–2017.

Variables	*N*	%
Age		
ays	174	20.7
28 days to < 1 year	254	30.2
1 to < 4 years	175	20.8
4 to < 7 years	99	11.8
7 years and up	138	16.5
Sex		
Male	447	53.2
Female	393	46.8
Child's city of origin		
Other municipalities	526	62.6
Hospital host municipality	311	37.0
Ignored	3	0.4
Chronic disease		
Yes	387	46.1
No	453	53.9
Nutritional diagnosis^a^		
Obesity/overweight	79	9.4
Malnutrition	236	28.1
Eutrophic	493	58.7
Ignored	32	3.8
Need for ventilatory assistance		
Yes	589	70.1
No	251	29.9
Need for drugs with vasoactive action		
Yes	200	23.8
No	639	76.1
Ignored	1	0.1
Probability of death: median (IIQ)^b^	4.2	(1.9–9.1)
Ignored	202	24.0
Healthcare‐related infection		
Yes	252	30.0
No	588	70.0
Death		
Yes	93	11.1
No	747	88.9

Abbreviation: IIQ, interquartile range.

^a^ Based on *z* weight‐for‐age score.

^b^ Based on PRISM‐II and CRIB scores.

The duration of stay in the PICU ranged from one to 147 days, with a median of 5 days (interquartile range,1–8 days). The length of hospital stay ranged from one to 600 days, with a median of 13 days (interquartile range:1–16 days). Two hundred fifty‐two children (30.0%) were diagnosed with HAIs during hospitalisation, and 93 (11.1%) did not survive (Table 1).

### 3.2 Late Nutrition

Late initiation of enteral nutrition was less frequent in the 28‐day to < 1‐year age group (RR:0.74; 95% CI:0.57–0.97) than in children aged 7 years or older, and was more frequent amongst children from other countries (RR:1.50; 95% CI:1.22–1.84), malnutrition (RR:1.23; 95% CI:1.02–1.49), and requiring vasoactive drugs in the first 24 h of hospitalisation (RR:1.58; 95% CI:1.33–1.88). Sex, chronic disease diagnosis, need for mechanical ventilation, and the probability of death were not associated with late initiation of enteral nutrition (Table 2).

**TABLE 2:** Bivariate analysis between late nutrition and demographic and clinical variables of Paediatric Intensive Care Unit (PICU) admissions, 2012–2017.

Variables	Total	Late nutrition			
	*N* = 840	*N* (%)	RR	95% CI	*p*
Age					
ays	174	77 (44.3)	1.05	0.81–1.36	0.695
28 days to < 1 year	254	79 (31.1)	**0.74**	**0.57–0.97**	**0.028**
1 to < 4 years	175	55 (31.4)	0.75	0.56–1.00	0.052
4 to < 7 years	99	42 (42.4)	1.01	0.75–1.37	0.952
7 years and up	138	58 (42.0)	1		
Sex					
Male	447	166 (37.1)	1.01	0.84–1.20	0.943
Female	393	145 (36.9)	1		
Child's city of origin					
Other municipalities	526	221 (42.0)	**1.50**	**1.22–1.84**	**< 0.001**
Hospital host municipality	311	87 (28.0)	1		
Chronic disease					
Yes	453	134 (34.6)	1.13	0.94–1.35	0.185
No	387	177 (39.1)	1		
Nutritional diagnosis^a^					
Obesity/overweight	79	20 (25.3)	0.72	0.49–1.07	0.107
Malnutrition	236	102 (43.2)	**1.23**	**1.02–1.49**	**0.031**
Eutrophy	493	173 (35.1)	1		
Need for ventilatory assistance					
Yes	458	225 (38.2)	1.12	0.91–1.36	0.290
No	208	86 (34.3)	1		
Need for a drug with vasoactive action					
Yes	200	103 (51.5)	**1.58**	**1.33–1.88**	**< 0.001**
No	639	208 (32.6)	1		
Probability of death: median (IIQ)^b^	638	4.2 (8.8)	1.002	0.997–1.007	0.50

Abbreviation: IIQ, interquartile range.

^a^ Based on *z* weight‐for‐age score.

^b^ Based on PRISM‐II and CRIB scores.

### 3.3 Risk Factors for Health Outcomes

Regarding the outcomes analysed, late initiation of enteral nutrition was associated with a longer stay in the PICU (*p* = 0.009), hospital stay (*p* < 0.001), incidence of HAIs (*p* < 0.001), and death (*p* = 0.017) (Table 3). Table 3 also shows other variables associated with the outcomes, especially the need for ventilatory assistance and vasoactive drugs, both of which were associated with all outcomes (*p* < 0.001).

**TABLE 3:** Bivariate analysis of late nutrition and adjustment variables with the outcomes of interest in a Paediatric Intensive Care Unit (PICU), 2012–2017.

Variables	Outcomes							
	PICU admission > 5 days		Hospital admissions > 13 days		HAIs		Death	
	*N* (%)	*p*	*N* (%)	*p*	*N* (%)	*p*	*N* (%)	*p*
Late nutrition								
Yes	173 (55.6)	**0.009**	177 (56.9)	**< 0.001**	121 (38.9)	**< 0.001**	45 (14.5)	**0.017**
No	246 (46.5)	—	232 (43.9)	—	131 (24.8)	—	48 (9.1)	—
**Age**								
0–27 days	99 (56.9)	**< 0.001**	94 (54.0)	**0.008**	44 (25.3)	0.669	11 (6.3)	**0.013**
28 days to < 1 year	150 (59.1)	**< 0.001**	141 (55.5)	**0.002**	92 (36.2)	**0.011**	34 (13.4)	0.617
1 to < 4 years	82 (46.9)	**0.013**	73 (41.7)	0.555	60 (34.3)	**0.036**	18 (10.3)	0.192
4 to < 7 years	43 (43.4)	0.087	48 (48.5)	0.119	24 (24.2)	0.850	9 (9.1)	0.597
7 years and up	45 (32.6)	—	53 (38.4)	—	32 (23.2)	—	21 (15.2)	—
Sex								
Male	214 (47.9)	0.918	212 (47.4)	0.946	119 (26.6)	**0.023**	41 (9.2)	0.063
Female	205 (52.2)	—	197 (50.1)	—	133 (33.8)	—	52 (13.2)	—
Child's city of origin								
Other municipalities	259 (47.9)	0.661	275 (52.3)	**0.012**	178 (33.8)	**0.002**	66 (12.5)	0.065
Hospital host municipality	158 (50.8)	—	134 (43.1)	—	73 (23.5)	—	26 (12.5)	—
Chronic disease								
Yes	196 (50.6)	0.682	195 (50.4)	0.362	141 (36.4)	**< 0.001**	65 (16.8)	**< 0.001**
No	223 (49.2)	—	214 (47.2)	—	111 (24.5)	—	28 (6.2)	—
Nutritional diagnosis*								
Obesity/overweight	28 (35.4)	**0.022**	32 (40.5)	0.447	14 (17.7)	0.054	5 (6.3)	0.278
Malnutrition	132 (55.9)	0.196	144 (61.0)	**< 0.001**	88 (37.3)	**0.019**	29 (12.3)	0.431
Eutrophy	251 (50.9)	—	223 (45.2)		142 (28.8)		51 (10.3)	
Need for ventilatory assistance								
Yes	374 (63.5)	**< 0.001**	319 (54.2)	**< 0.001**	214 (36.3)	**< 0.001**	83 (14.1)	**< 0.001**
No	45 (17.9)	—	90 (35.9)	—	38 (15.1)	—	10 (4.0)	—
Need for a drug with vasoactive action								
Yes	130 (65.0)	**< 0.001**	120 (60.0)	**< 0.001**	96 (48.0)	**< 0.001**	47 (23.5)	**< 0.001**
No	289 (45.2)	—	288 (45.1)	—	156 (24.4)	—	56 (9.8)	—
Probability of death: median (IIQ)^a^	5.1 (2.2–11.0)	0.075	4.2 (2.2–11.0)	**0.042**	6.2 (2.6–15.7)	**< 0.001**	12.2 (3.4–39.3)	**< 0.001**

Abbreviations: HAI, healthcare‐related infection; IIQ, interquartile range; PICU, paediatric intensive care unit.

^a^ By means of PRISM‐II and CRIB scores.

### 3.4 Late Nutrition and Health Outcomes

After all adjustments by Poisson multiple regression, late initiation of enteral nutrition remained a significant risk factor for a length of stay for more than 5 days in the PICU (RR:1.15; 95% CI:1.01–1.31), for over 13 days in the hospital (RR:1.22; 95% CI:1.05–1.43), and for the incidence of healthcare‐related infections (RR:1.33; 95% CI:1.06–1.66). Although the incidence of death was higher in the group in which nutrition was started late (14.5%) than in those with early nutrition (9.5%), this association was not statistically significant after adjusting for severity indicators (Model 2) (Table 4). Owing to the proportion of information loss in the final statistical model (Model 2), mainly because of missing information on severity scores at admission (24%; Table 1), the associations between delayed nutrition and the outcomes analysed were compared between cases with and without information. Amongst the losses, the associations were stronger than those observed in the cases with information. Thus, if these losses could be included in the regression models, the relative risk estimates would be even higher than those observed.

**TABLE 4:** Crude and adjusted analysis for outcomes in children receiving late nutrition in a Paediatric Intensive Care Unit (PICU), 2012–2017.

Outcomes	Raw analysis RR (95% CI)	Adjusted analysis (Model 1) RR (95% CI)	Adjusted analysis (Model 2) RR (95% CI)
PICU admission > 5 days	**1.20 (1.05–1.37)**	**1.19 (1.04–1.36)** ^**a**^	**1.15 (1.01–1.31)** ^**e**^
Hospital admission > 13 days	**1.30 (1.13–1.49)**	**1.27 (1.11–1.46)** ^**b**^	**1.22 (1.06–1.43)** ^**e**^
HAIs	**1.57 (1.28–1.93)**	**1.54 (1.25–1.90)** ^**c**^	**1.33 (1.06–1.66)** ^**e**^
Death	**1.60 (1.09–2.34)**	**1.59 (1.08–2.35)** ^**d**^	1.11 (0.73–1.87)^e^

Abbreviations: HAIs, healthcare‐related infections; PICU, paediatric intensive care unit.

^a^ Adjusted by age and nutritional diagnosis.

^b^ Adjusted for age, child's city of origin and nutritional diagnosis.

^c^ Adjusted for age, sex, child's city of origin, chronic disease and malnutritional diagnosis.

^d^ Adjusted for age, sex, child's city of origin and chronic disease.

^e^ Adjusted for Model 1 variables, plus the need for ventilatory assistance or vasoactive drugs, and the probability of death.

### 3.5 Survival Analysis of Health Outcomes

**FIGURE 1:**
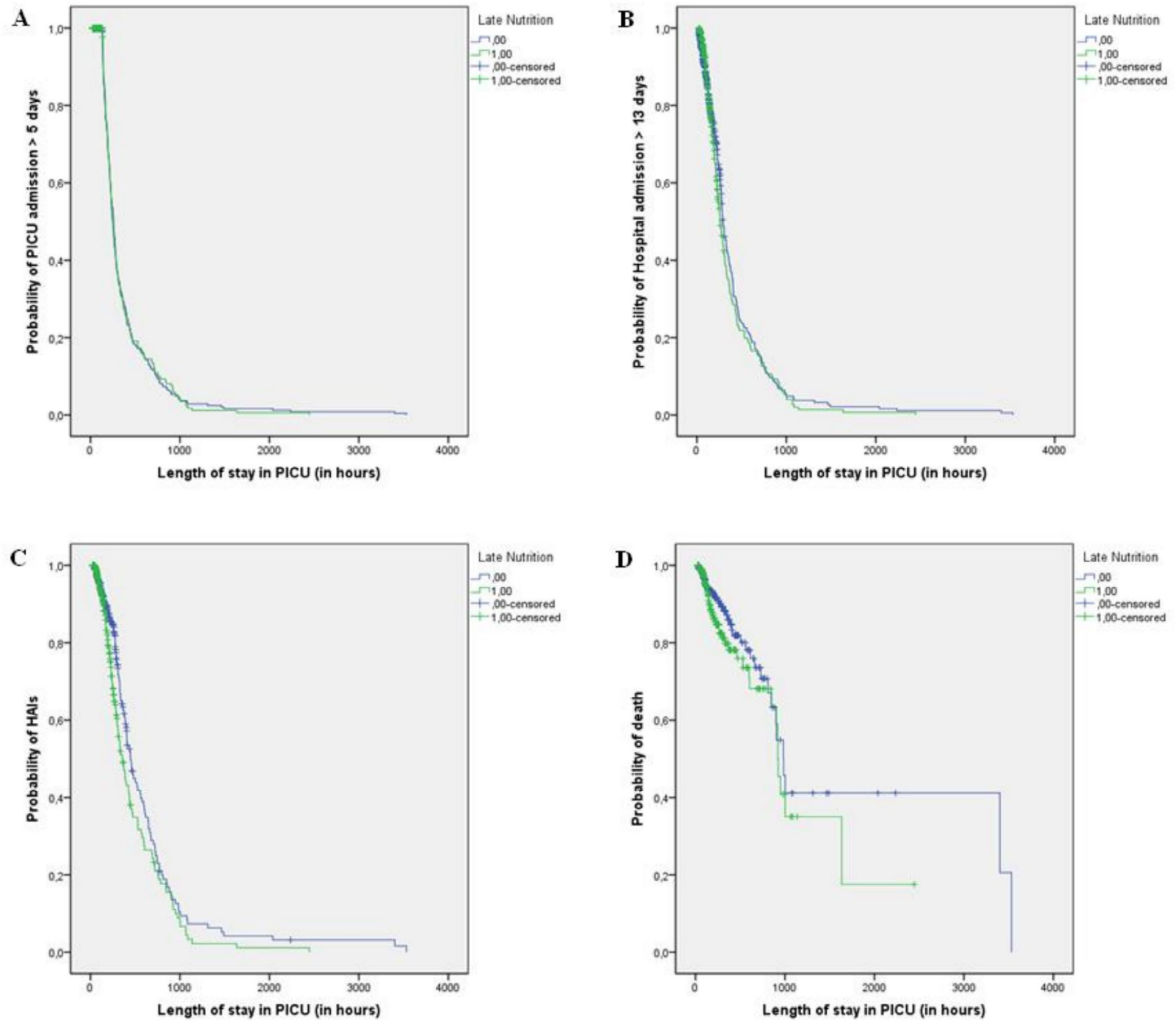
Probability of PICO admission for more than 5 days (A), hospital admission for more than 13 days (B), healthcare‐associated infections (HAIs) (C), and death (D) due to delayed nutrition, considering PICO length of stay (in hours), Paediatric Intensive Care Unit (PICU), 2012–2017.

In Figure 1, when examining the probability of hospitalisation in PICO for more than 5 days (A), hospitalisation for more than 13 days (B), healthcare‐associated infections (HAIs) (C), and death (D) using the Kaplan–Meier curve, it was observed that only HAIs showed a statistically significant difference (*p* = 0.021), with a Log Rank of 5.348. Hospitalisation in PICO for more than 5 days (Log Rank 0.10; *p* = 0.921), hospitalisation for more than 13 days (Log Rank 1.109; *p* = 0.292), and death (Log Rank 2.263; *p* = 0.132) did not show significant results.

## 4 Discussion

This study found that children aged 28 days to less than 1 year were fed earlier. After all statistical adjustments, patients who received delayed nutrition had longer stays in the PICU and hospital, and a higher risk of HAIs. Regarding death, there was no significant difference between the groups after adjusting for the severity markers at admission.

Similar age‐related results were obtained in a North American study [12], in which the median ages of the groups fed earlier and later were 0.7 and 4 years, respectively. It is believed that children under 1 year of age need nutrition earlier because they have higher energy requirements for growth [13]. This study did not find an association between late initiation of nutrition and the need for ventilatory assistance in the first 24 h after admission, which may be explained by the large proportion of children aged less than 1 year in the sample, an age group associated with earlier initiation of nutrition and greater need for mechanical ventilation [14].

A multicenter study conducted in the US [15] revealed that children with worse severity scores or those who required ventilatory support later received nutrition. In the present study, delayed initiation of nutrition was significantly associated with the use of vasoactive drugs in the first 24 h and with higher odds of death, which corroborated the correlation of delayed nutritional support in patients with more severe conditions.

A South Korean study found an association between malnutrition and early onset of nutrition [16]. The present study observed contradictory results with a significant association between malnutrition and late‐onset nutrition. Likewise, malnourished children from other municipalities also received nutrition later, which may be related to the severity at admission, considering that the distance from the hospital and problems in transportation [17] contribute to admission in worse vitality conditions [18, 19]. In this study, it was not considered that, possibly, the late start of feeding may be related to the severity upon admission, or even during the first days of hospitalisation, and not necessarily as an intensive treatment option, which certainly had an impact on the patient's health. Situations such as haemodynamic instability, multiple traumas and digestive bleeding were the factors that limited the initiation of feeding and were not a decision by the care team.

The association between delayed nutrition and longer PICU and hospital stays is consistent with the results of a clinical trial of 608 children with hyperglycemia on mechanical ventilation who were receiving vasoactive drugs [4]. Another study of 5105 North American children found no association between early nutrition and length of stay in the PICU after adjustment for age, severity score, and study centre [15]. This discrepancy may be due to methodological differences, because in this North American study [15], children younger than 30 days were excluded, and an age group in which late initiation of nutrition showed greater harm. Starting enteral nutrition earlier for children hospitalised with serious diseases allows nutritional goals to be achieved earlier, especially in younger children, such as premature newborns, contributing to reduced hospital stays [20].

The initiation of enteral nutrition after 24 h was a risk factor for acquiring HAIs in this study. The same was observed in a study of children with hyperglycemia [4], with a higher incidence of HAIs in patients whose nutrition was started 48 h after hospitalisation. Although these infections are common outcomes in certain age groups or children diagnosed with chronic diseases, such as malnutrition [21, 22], the association between delayed nutrition and HAIs in the present study remained significant even after adjusting for malnutrition and severity at admission.

Although mortality was higher in the group with delayed nutrition (14.5% vs. 9.5%), this association was not significant when the final model was adjusted for the need for ventilatory assistance or vasoactive drugs and probability of death. In a study conducted in Turkey, late initiation of enteral nutrition was associated with higher mortality [8]. A recent North American study of children with traumatic brain injury revealed no difference in mortality when comparing the times of nutrition initiation [15]. However, late initiation was independently associated with mortality in a subanalysis of patients with more severe conditions. Mortality was also significantly lower in the early nutrition group in studies conducted in South Korea [16] and the United States [9]. In this study, despite the non‐statistical significance, the clinical correlation between the late start of the diet and mortality must be valued, considering that, indirectly, by increasing the length of stay in the PICU and in the hospital, by increasing the time of ventilatory assistance or the incidence of healthcare‐related infections, the longer fasting time had an impact on the survival rate. Eventually, some variables selected as confounders may also have interfered with statistical power.

There is still no definitive consensus on practises for starting digestive feeding in children admitted to the PICU. On the other hand, there are still divergences between the different sectors, whether in relation to the age group or the profile of the critically ill patient. However, there is a certain tendency to start nutritional support earlier via enteral or oral routes, especially in children under 1 year of age. With certain malformations of the gastrointestinal system and digestive haemorrhages as exceptions, those responsible for direct assistance to critically ill children must give nutritional support the same priority that already exists in relation to ventilatory, haemodynamic support and infectious control.

The research was based on medical records, which often present problems such as the absence of information. In cohort studies, all participants (exposed and unexposed) must be at risk of developing the trained stages. Any mistakes in sample selection may compromise accuracy. In this sense, there was a concern to exclude patients suspected of having already suffered advanced diseases or those with the potential for early evolution, for example, death within the first 24 h of admission. The option to dichotomize the time to start the diet is because it is a specific and measurable exposure factor. In addition, the study's retrospective design hindered the identification of the number of calories or proteins in the initial prescription and the route of administration used, or even the total number of interruptions, a common practise in the care of critically ill children [23, 24]. Despite these limitations, this study included a relatively large number of children, which contributed to the statistical power of association analyses. Another characteristic of this study was that the population was composed of children with a mixed profile of causes of hospitalisation, covering different age groups and a variety of comorbidities and clinical and surgical diseases that are common in low‐ and middle‐income countries. Considering these limitations, it is believed that randomised clinical trials, with objective control of the sample exposure factor, and more careful selection of participants can contribute to more robust evidence.

Although no association between delayed nutrition and mortality was observed, the results of this study indicate that delaying the initiation of nutrition can increase the length of stay in the PICU and hospital, and the incidence of healthcare‐related infections. These outcomes prolong hospitalisation and increase the physical and emotional consequences for hospitalised children and their families. Additionally, they may reduce access to intensive care for other children who need beds. Further studies are required to identify the causes of delayed nutrition [13], especially those related to the care of children with serious conditions. By addressing these causes, we may contribute to better outcomes for hospitalised children, lower in‐hospital morbidity, and greater rotation of PICU beds, providing greater access to highly complex care in countries that still lack specialised beds for this type of care.

Based on the observed results, it is important to explore specific barriers to the timely initiation of enteral nutrition within the context of Brazilian PICUs. Furthermore, future research should investigate the impact of nutritional timing on other important outcomes, such as growth. Finally, developmental delays should be assessed, and interventions aimed at improving enteral nutrition practises, as well as their cost‐effectiveness in PICUsshould be evaluated.

## Author Contributions

A.L.J. and F.L.G. contributed to the design and implementation of the research, E.G. and A.M.R.S. to the analysis of the results and to the writing of the manuscript. S.M.A. conceived and supervised the project.

## Ethics Statement

This study was approved by the Research Ethics Committee of the State University of Londrina, Paraná, Brazil.

## Conflicts of Interest

The authors declare no conflicts of interest.

## Data Availability

The data used in the study were obtained from medical records made available by the hospital's archive sector.

## References

[1] K. Joosten , D. Kerklaan , and S. Verbruggen , “Nutritional Support and the Role of the Stress Response in Critically Ill Children,” Current Opinion in Clinical Nutrition and Metabolic Care 19, no. 3 (2016): 226–233. 26963579 10.1097/MCO.0000000000000268

[2] F. V. Valla , D. K. Young , M. Rabilloud , et al., “Thigh Ultrasound Monitoring Identifies Decreases in Quadriceps Femoris Thickness as a Frequent Observation in Critically Ill Children,” Pediatric Critical Care Medicine 18, no. 8 (2017): e339–e347. 28650903 10.1097/PCC.0000000000001235

[3] F. V. Valla , J. Berthiller , B. Gaillard‐Le‐Roux , et al., “Faltering Growth in the Critically Ill Child: Prevalence, Risk Factors, and Impaired Outcome,” European Journal of Pediatrics 177, no. 3 (2018): 345–353. 29243190 10.1007/s00431-017-3062-1

[4] V. Srinivasan , N. R. Hasbani , N. M. Mehta , et al., “Early Enteral Nutrition Is Associated With Improved Clinical Outcomes in Critically Ill Children: A Secondary Analysis of Nutrition Support in the Heart and Lung Failure‐Pediatric Insulin Titration Trial,” Pediatric Critical Care Medicine 21, no. 3 (2020): 213–221. 31577692 10.1097/PCC.0000000000002135 PMC7060827

[5] S. Edwardson and C. Cairns , “Nosocomial Infections in the ICU,” Anaesth Intensive Care Med 20, no. 1 (2019): 14–18.

[6] T. Hatachi , Y. Inata , and K. Moon , “Effects of Healthcare‐Associated Infections on Length of PICU Stay and Mortality,” Pediatric Critical Care Medicine 20, no. 11 (2019): e503–e509. 31415445 10.1097/PCC.0000000000002096

[7] L. N. Tume , F. V. Valla , K. Joosten , et al., “Nutritional Support for Children During Critical Illness: European Society of Pediatric and Neonatal Intensive Care (ESPNIC) Metabolism, Endocrine and Nutrition Section Position Statement and Clinical Recommendations,” Intensive Care Medicine 46, no. 3 (2020): 411–425. 32077997 10.1007/s00134-019-05922-5 PMC7067708

[8] S. Baǧci , E. Keleş , F. Girgin , et al., “Early Initiated Feeding Versus Early Reached Target Enteral Nutrition in Critically Ill Children: An Observational Study in Paediatric Intensive Care Units in Turkey,” Journal of Paediatrics and Child Health 54, no. 5 (2018): 480–486. 29278447 10.1111/jpc.13810

[9] T. A. Mikhailov , S. J. Gertz , E. M. Kuhn , et al., “Early Enteral Nutrition Is Associated With Significantly Lower Hospital Charges in Critically Ill Children,” JPEN Journal of Parenteral and Enteral Nutrition 42, no. 5 (2018): 920–925. 30001462 10.1002/jpen.1025

[10] N. M. Mehta , H. E. Skillman , S. Y. Irving , et al., “Guidelines for the Provision and Assessment of Nutrition Support Therapy in the Pediatric Critically Ill Patient: Society of Critical Care Medicine and American Society for Parenteral and Enteral Nutrition,” JPEN Journal of Parenteral and Enteral Nutrition 41, no. 5 (2017): 706–742. 28686844 10.1177/0148607117711387

[11] M. M. Pollack , U. E. Ruttimann , and P. R. Getson , “Pediatric Risk of Mortality (PRISM) Score,” Critical Care Medicine 16, no. 11 (1988): 1110–1116. 3048900 10.1097/00003246-198811000-00006

[12] T. A. Mikhailov , E. M. Kuhn , J. Manzi , et al., “Early Enteral Nutrition Is Associated With Lower Mortality in Critically Ill Children,” JPEN Journal of Parenteral and Enteral Nutrition 38, no. 4 (2014): 459–466. 24403379 10.1177/0148607113517903

[13] L. N. Tume , F. V. Valla , A. A. Floh , et al., “Priorities for Nutrition Research in Pediatric Critical Care,” JPEN Journal of Parenteral and Enteral Nutrition 43, no. 7 (2019): 853–862. 30588643 10.1002/jpen.1498

[14] A. Haney , E. Burritt , and C. J. Babbitt , “The Impact of Early Enteral Nutrition on Pediatric Acute Respiratory Failure,” Clinical Nutrition ESPEN 26 (2018): 42–46. 29908681 10.1016/j.clnesp.2018.04.017

[15] M. F. Canarie , S. Barry , C. L. Carroll , et al., “Risk Factors for Delayed Enteral Nutrition in Critically Ill Children,” Pediatric Critical Care Medicine 16, no. 8 (2015): e283–e289. 26237658 10.1097/PCC.0000000000000527 PMC4592402

[16] H. Lee , S. O. Koh , H. Kim , M. H. Sohn , K. E. Kim , and K. W. Kim , “Avoidable Causes of Delayed Enteral Nutrition in Critically Ill Children,” Journal of Korean Medical Science 28, no. 7 (2013): 1055–1059. 23853489 10.3346/jkms.2013.28.7.1055 PMC3708077

[17] B. Balakrishnan , K. T. Flynn‐O'Brien , P. M. Simpson , M. Dasgupta , and S. J. Hanson , “Enteral Nutrition Initiation in Children Admitted to Pediatric Intensive the Impact of Early Enteral Nutrition on Pediatric Acute Respiratory Failure. Clin Nutr ESPEN Care Units After Traumatic Brain Injury,” Neurocritical Care 30, no. 1 (2019): 193–200. 30171446 10.1007/s12028-018-0597-6

[18] F. A. Moore , “The Role of the Gastrointestinal Tract in Postinjury Multiple Organ Failure,” American Journal of Surgery 178, no. 6 (1999): 449–453. 10670850 10.1016/s0002-9610(99)00231-7

[19] R. S. Goldwasser , M. S. Lobo , E. F. de Arruda , et al., “Difficulties in Access and Estimates of Public Beds in Intensive Care Units in the State of Rio de Janeiro,” Revista de Saúde Pública 50 (2016): 19, 10.1590/S1518-8787.2016050005997. 27191155 PMC4902093

[20] A. Yock‐Corrales , N. Casson , G. Sosa‐Soto , and R. A. Orellana , “Pediatric Critical Care Transport: Survey of Current State in Latin America. Latin American Society of Pediatric Intensive Care Transport Committee,” Pediatric Emergency Care 38, no. 1 (2022): e295–e299. 33105465 10.1097/PEC.0000000000002273

[21] A. Kawaguchi , C. C. Nielsen , G. Guerra , et al., “Epidemiology of Pediatric Critical Care Transport in Northern Alberta and the Western Arctic,” Pediatric Critical Care Medicine 19, no. 6 (2018): e279–e285. 29406372 10.1097/PCC.0000000000001491

[22] B. Sahiledengle , F. Seyoum , D. Abebe , et al., “Incidence and Risk Factors for Hospital‐Acquired Infection Among Paediatric Patients in a Teaching Hospital: A Prospective Study in Southeast Ethiopia,” BMJ Open 10, no. 12 (2020): e037997. 10.1136/bmjopen-2020-037997 PMC7747586 33334828

[23] C. Culpepper , K. Hendrickson , S. Marshall , J. Benes , and T. R. Grover , “Implementation of Feeding Guidelines Hastens the Time to Initiation of Enteral Feeds and Improves Growth Velocity in Very Low Birth‐Weight Infants,” Advances in Neonatal Care 17, no. 2 (2017): 139–145. 27750266 10.1097/ANC.0000000000000347

[24] F. M. Silva , A. C. Bermudes , I. R. Maneschy , et al., “Impact of Early Enteral Nutrition Therapy on Morbimortality Reduction in a Pediatric Intensive Care Unit: A Systematic Review,” Rev Assoc Med Bras 59, no. 6 (2013): 563–570, 10.1016/j.ramb.2013.06.013. 24199586

